# Suspected Simple Appendicitis in Children: Should We Use a Nonoperative, Antibiotic-Free Approach? An Observational Study

**DOI:** 10.3390/children11030340

**Published:** 2024-03-13

**Authors:** Patricia Reis Wolfertstetter, John Blanford Ebert, Judith Barop, Markus Denzinger, Michael Kertai, Hans J. Schlitt, Christian Knorr

**Affiliations:** 1 Faculty of Medicine, University Medical Center Regensburg, Franz-Josef-Strauss-Allee 11, 93053 Regensburg, Germany; 2 Department of Psychiatry and Psychotherapy, Medbo Bezirksklinikum Regensburg, Universitaetsstrasse 84, 93053 Regensburg, Germany; 3 Department of Pediatric Surgery and Pediatric Orthopedics, Hospital St. Hedwig of the Order of St. John of God, University Children’s Hospital Regensburg (KUNO), Steinmetzstrasse 1-3, 93049 Regensburg, Germany; 4 Section of Pediatric Orthopedics, Department of Orthopedics and Trauma Surgery, Hospital St. Marien Amberg, Mariahilfberg 7, 92224 Amberg, Germany; 5 Department of Surgery, University Hospital Regensburg, Franz-Josef-Strauss-Alee 11, 93053 Regensburg, Germany

**Keywords:** abdominal pain, appendicitis, simple, conservative treatment, appendectomy, pediatrics

## Abstract

Background: Simple appendicitis may be self-limiting or require antibiotic treatment or appendectomy. The aim of this study was to assess the feasibility and safety of a nonoperative, antibiotic-free approach for suspected simple appendicitis in children. Methods: This single-center, retrospective study included patients (0–17 years old) who were hospitalized at the pediatric surgery department due to suspected appendicitis between 2011 and 2012. Data from patients who primarily underwent appendectomy were used as controls. The follow-up of nonoperatively managed patients was conducted in 2014. The main outcome of interest was appendicitis recurrence. Results: A total of 365 patients were included: 226 were treated conservatively and 139 underwent appendectomy. Fourteen (6.2% of 226) of the primarily nonoperatively treated patients required secondary appendectomy during follow-up, and histology confirmed simple, uncomplicated appendicitis in 10 (4.4% of 226) patients. Among a subset of 53 patients managed nonoperatively with available Alvarado and/or Pediatric Appendicitis Scores and sonographic appendix diameters in clinical reports, 29 met the criteria for a high probability of appendicitis. Three of these patients (10.3% of 29) underwent secondary appendectomy. No complications were reported during follow-up. Conclusions: A conservative, antibiotic-free approach may be considered for pediatric patients with suspected uncomplicated appendicitis in a hospital setting. Only between 6 and 10% of these patients required secondary appendectomy. Nevertheless, the cohort of patients treated nonoperatively was likely to have also included individuals with further abdominal conditions other than appendicitis. Active observation and clinical support during the disease course may help patients avoid unnecessary procedures and contribute to spontaneous resolution of appendicitis or other pediatric conditions as the cause of abdominal pain. However, further studies are needed to define validated diagnostic and management criteria.

## 1. Introduction

Acute appendicitis is one of the most common abdominal conditions in childhood and adolescence. Approximately one-third of the indications for inpatient admission for abdominal pain in the pediatric population are suspected appendicitis [1]. Appendectomy has been performed for more than 130 years as standard therapy for acute appendicitis [2,3]. Recently, the role of appendectomy as a generally unique valid standard for the treatment of suspected appendicitis has been frequently questioned. In addition to standard operative intervention, conservative therapy has been the subject of a considerable number of studies in adults and, increasingly, children [3,4,5,6,7,8,9,10,11]. Current studies have demonstrated comparable outcomes between nonoperative therapy with antibiotics and the operative approach for uncomplicated appendicitis in children [6,12]; however, for conservative management, the recurrence rate after one year is approximately 32% [9].

The cause of approximately 60% of appendicitis cases remains unclear [13,14]. Luminal obstruction (e.g., by fecalith, lymphoid hyperplasia, or adhesions; rarely, by parasites, tumors, or foreign bodies) is responsible for the remaining cases [13,15], although these features can also be present in the absence of inflammation [14,16]. The presence of an appendicolith in imaging studies has been described as a possible risk factor for appendicitis recurrence and treatment failure, so conservative therapy is not recommended for these patients [8,11,13]. Furthermore, observations of the natural history of the disease suggest that uncomplicated and incipient appendicitis can be self-limiting and can occur in combination with other inflammatory conditions [3,17,18,19,20,21].

In cases of suspected appendicitis in children, hospital admission and clinical monitoring are recommended. Initially, nonoperative therapy includes parenteral fluids, analgesics, and, if necessary, appropriate laxatives and antibiotics [3,6,18,22,23,24]. Active observation of the disease course, including clinical re-evaluations and repeated ultrasound (US), as well as the control of biochemical inflammation parameters, can be used to support the maintenance of conservative treatment or escalation to a surgical approach. This strategy may contribute to the spontaneous resolution of uncomplicated, incipient, or early cases of appendicitis, avoiding invasive procedures and complications [17,19,25].

The purpose of this observational study was to investigate the feasibility and safety of a nonoperative, antibiotic-free approach for pediatric patients presenting with suspected uncomplicated appendicitis.

## 2. Materials and Methods

### 2.1. Study Design, Population, and Parameters

We conducted a retrospective electronic data analysis of inpatients aged between 0 and 17 years who were hospitalized with suspected appendicitis at the department of pediatric surgery in a single tertiary medical center (Pediatric Hospital St. Hedwig in Regensburg, Germany) during the 2-year period from 1 January 2011 to 31 December 2012. The study was approved by the Ethics Committee of the University of Regensburg (no. 19-1339-104; date of approval: 20 February 2019). The participants were retrospectively divided into two groups: primarily conservative or primarily operative treatment. Primary or immediate operation was defined by appendectomy (conventional, laparoscopic, or single port) during the first/index hospital admission due to suspected appendicitis. Primary conservative therapy was defined by supportive care, including the administration of parenteral fluids, by initially pausing or decreasing oral food intake and, if necessary, providing clysters and analgesics, with no use of antibiotics. The decision between nonoperative, antibiotic-free management, and appendectomy was made clinically by a consultant pediatric surgeon.

After chart review, documented parameters in both groups at admission were recorded, which included the following:

▪ Patient demographic data: Age and sex.
▪ Clinical signs and symptoms: Nausea or vomiting, right lower quadrant (RLQ) tenderness, rebound pain, and fever.
▪ Ultrasonographic findings: Free intraabdominal fluid, visible appendix, appendix diameter, and appendicolith.
▪ Biochemical parameters: Leukocytosis, C-reactive protein (CRP) elevation, and neutrophilia.

The length of hospital stay was also assessed for both groups.

To assess the likelihood of appendicitis in pediatric patients treated conservatively, two validated scores, the Alvarado Score (AS) and the Pediatric Appendicitis Score (PAS) (Table 1), were retrospectively assessed as supportive diagnostic instruments [26,27]. Due to data availability, scores were calculated only for patients treated nonoperatively.

The histopathological findings were recorded for patients who underwent appendectomy.

The parents and/or patients in the conservative group were interviewed in the context of prospective clinical follow-up after discharge in 2014 so that patient outcomes could be evaluated after a minimum of 1 year. Patients completed a survey regarding recurrent abdominal pain during the first 3 months after discharge, new hospital admission due to suspected appendicitis, and another conservative therapy or appendectomy (if present, medical records and histology were assessed) until the time of follow-up. Those data were then analyzed retrospectively.

### 2.2. Inclusion and Exclusion Criteria

Only children admitted for inpatient treatment were included in the study. Patients who presented to the emergency department or outpatient clinic with abdominal pain and who were discharged after initial evaluation and management without hospital admission were excluded from the analysis. In this patient cohort, no antibiotics were prescribed to treat patients with suspected appendicitis. Patients that received antibiotics due to concurrent disease (e.g., pneumonia) were excluded from the analysis. The included patients from the conservative group had also not previously received antibiotics specifically for appendicitis treatment. Previous antibacterial treatment (due to another disease) prior to the index hospital admission was not assessed. The inclusion and exclusion criteria are summarized in Table 2.

The surgical group with uncomplicated appendicitis was included as a diagnostic control for conservative patients, allowing for the comparison of demographic, clinical, biochemical, and sonographic parameters between the two populations. Patients with histologically normal appendices (negative appendectomies) were excluded because the controls should be patients with confirmed appendicitis. 

### 2.3. Subgroup of Nonoperatively Treated Patients

To carry out a closer analysis of the patients initially treated conservatively and without antibiotics, a subgroup of patients with available appendicitis scores (AS and/or PAS) and US reports with a visible appendix and appendix diameter was assessed. As in previous research, a valid diagnostic approach included clinical assessment with appendicitis scores combined with imaging studies [28,29]. In the proposed clinical pathway, patients with low-risk Alvarado and/or Pediatric Appendicitis Scores were considered unlikely to have appendicitis. The data from patients with intermediate scores were combined with appendix ultrasound data and the patients were classified as having appendicitis if their appendix diameter was ≥6 mm. Patients with high risk scores were also classified as having appendicitis. The classification goal was to identify individuals with a very high probability of actually having acute appendicitis.

### 2.4. Outcomes of Interest

The primary outcome of interest was the recurrence of appendicitis, which was determined via appendectomy with histological confirmation of acute appendicitis as follows: short-term, within 6 months and 6–12 months after discharge; or long-term, after 12 months. The secondary outcomes included the length of primary hospitalization, recurrent hospitalization due to suspected appendicitis, recurrent abdominal pain within 3 months after discharge, and complications after conservative management.

### 2.5. Statistical Analysis

Descriptive statistics were calculated using the number of events and percentages. Comparisons between groups were performed using chi-squared tests and Fisher’s exact tests for categorical variables. To test the normality of the data distribution, the Kolmogorov–Smirnov test was used. For continuous variables with a normal distribution, a *t* test was used to compare the mean values. For nonnormally distributed variables, the Mann–Whitney U test was used to compare the median values. *p* < 0.05 indicated statistical significance. All of the statistical analyses were performed using IBM SPSS Statistics 25 software for Windows.

## 3. Results

### 3.1. Study Population

**Figure 1:**
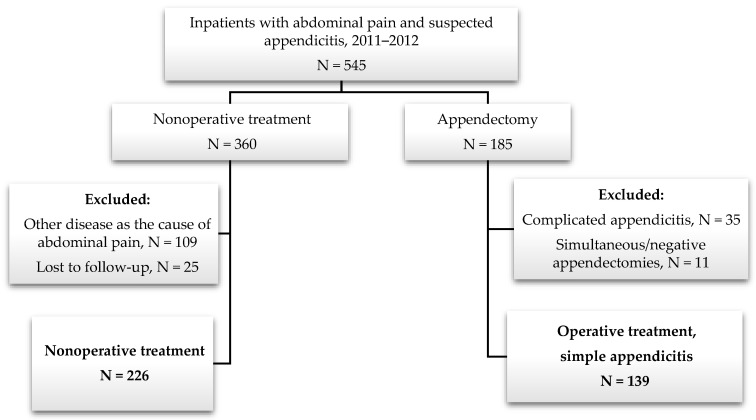
General study population flowchart.

A total of 545 patients with abdominal pain and suspected appendicitis were admitted to the pediatric surgery department during the two-year period. A total of 360 children were primarily treated nonoperatively and 185 underwent immediate appendectomy (i.e., during the index hospitalization). A total of 365 patients (67%) met the inclusion criteria—226 (62%) in the nonoperative group and 139 (38%) in the operative group (Figure 1).

#### Nonoperative Treatment: Subgroup with Available Appendicitis Scores and Appendix Diameter on US

**Figure 2:**
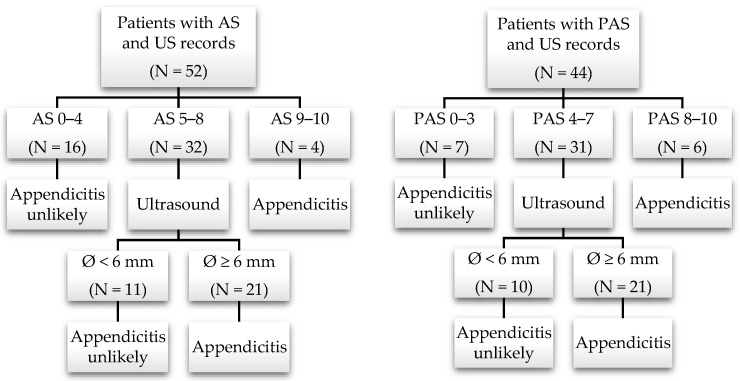
Flowcharts and patient subgroup classifications. (**left**) Alvarado Score (AS); (**right**) Pediatric Appendicitis Score (PAS). US, ultrasound. The appendix diameter is displayed as the mean (SD): appendicitis unlikely, n = 24: 5.5 mm (SD 2.2); appendicitis, n = 29: 7.5 mm (SD 2.2), *p* < 0.001.

A total of 53 patients in the primarily nonoperative group (n = 226) had at least one available appendicitis score (AS and/or PAS) and a visible appendix on US in clinical reports. The retrospectively defined criteria for diagnosing acute appendicitis included, as stated in the Methods section, a positive appendicitis score (Alvarado 9–10 and/or PAS 8–10) or an appendix diameter of at least 6 mm on US combined with an intermediate score (Alvarado 5–8 and/or PAS 4–7), as shown in Figure 2. Based on the abovementioned criteria, appendicitis was diagnosed in a total of 29 patients (54.7% of 53).

### 3.2. Population Characteristics and Parameters

The characteristics and variable distributions of the included children who underwent nonoperative therapy and primary appendectomy are summarized in Table 3. A comparison of the operative group with the complete study population of the nonoperative group is shown in the Appendectomy column (n = 139 vs. Nonoperative Treatment group; n = 226; *p*-value ^§^). For the comparison of parameters between the nonoperative subgroup (n = 29) and primary surgery patients, the data are shown in the columns Appendectomy, n = 139 vs. Subgroup Nonoperative Treatment, n = 29, and *p* value ^§§^.

Patient demographic data such as age and sex were comparable between the surgical and conservative groups since the differences were not statistically significant. For the general population, significant differences were observed in the clinical, laboratory, and sonographic parameters. When comparing the variables of the subgroup of 29 patients with those of the operated cohort, no significant differences were found in terms of leukocytosis, elevated CRP levels, nausea/vomiting, RLQ tenderness, rebound pain, the presence of free abdominal fluid, or appendix diameter on US.

The variable appendicolith (calcified material within the appendix) was poorly documented in the US records. This variable was mentioned in only 7 US reports: 2 patients had a positive sign, and 5 patients had no signs of appendicolith. All 5 patients without appendicolith were treated conservatively with no recurrence in follow-up. One patient with appendicolith underwent primary operation (histologically phlegmonous appendicitis), and the other patient underwent a nonoperative approach without recurrence during follow-up. Due to missing variables, performing statistical analysis and comparisons for this variable was not feasible.

Patients treated nonoperatively had a median score of 5 for both AS and PAS (interquartile range [IQR] 3–6 and 4–6, respectively). With respect to the nonoperative subgroup (n = 29), the median score was 6.5 (IQR 5.2–8) for AS and 6 (IQR 4–7) for PAS.

The histological findings of patients who underwent primary appendectomy are summarized in Table 4.

### 3.3. Outcomes

#### 3.3.1. Length of Hospital Stay during Index Hospitalization

Patients who underwent nonoperative therapy had a significantly shorter hospital stay (mean of 1.9 days) than those in the operative group (4.5 days), *p* = 0.000 (Table 3). In addition to the recovery time necessary after a surgical procedure, those patients may have experienced a longer hospital stay for observation prior to the indication for surgery.

#### 3.3.2. Follow-Up after Nonoperative Treatment: Secondary Appendectomies, Recurrent Abdominal Pain, and Complications

**Figure 3:**
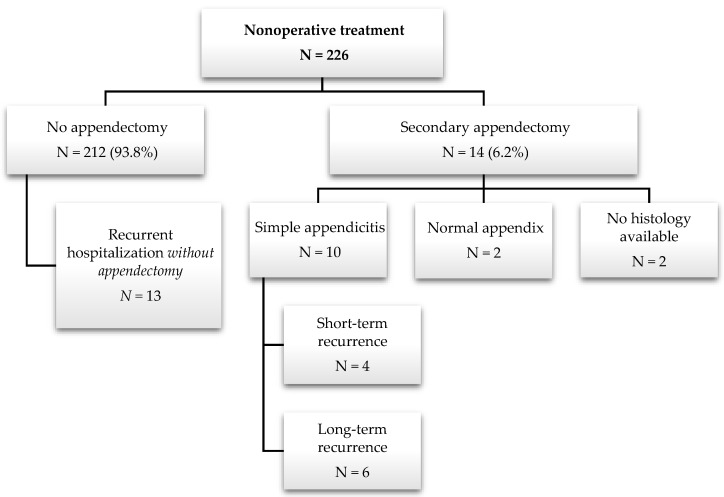
Flowchart of follow-up after nonoperative treatment.

Follow-up of the initially nonoperatively treated patients (n = 226) was performed in 2014 (minimum of 1.3 years and maximum of 4.3 years after primary treatment). Fourteen patients (6.2% of 226), nine boys and five girls, underwent secondary appendectomy after discharge following nonoperative therapy and readmission (Figure 3). Ten surgical procedures were performed at external hospitals, and four were performed at Children’s Hospital St. Hedwig. All secondary appendectomies were performed during the first hospital readmission after conservative treatment.

Ten (4.4% of 226) secondarily operated patients were histologically confirmed to have simple appendicitis (six at external hospitals and four at Children’s Hospital St. Hedwig), as shown in Table 5.

In patients with histologically confirmed appendicitis (acute and subacute, n = 10), appendectomy was performed a mean of 13.9 months after initial discharge (minimum of 5 days and maximum of 23.2 months). In summary, short-term recurrence was observed in four patients (1.8% of 226), with a mean of 6.2 months: one patient underwent appendectomy 5 days after initial discharge due to early pain recurrence, and three patients underwent appendectomy within 6–12 months (7.7, 7.9, and 8.9 months). Six patients (2.7% of 226) experienced long-term recurrence (after 12 months), with a mean of 18.9 months, a minimum of 16.2 months, and a maximum of 23.2 months. Negative appendectomies (normal appendix on histology) were performed 14.7 and 36.1 months after initial discharge.

Thirteen patients (5.7% of 226) in the nonoperative group underwent recurrent hospitalization (once to four times) without appendectomy due to recurrent abdominal pain. Forty-nine patients (21.6% of 226) reported relevant abdominal pain during the first 3 months after undergoing conservative therapy.

No patients experienced any complications or perforation during follow-up.

**Figure 4:**
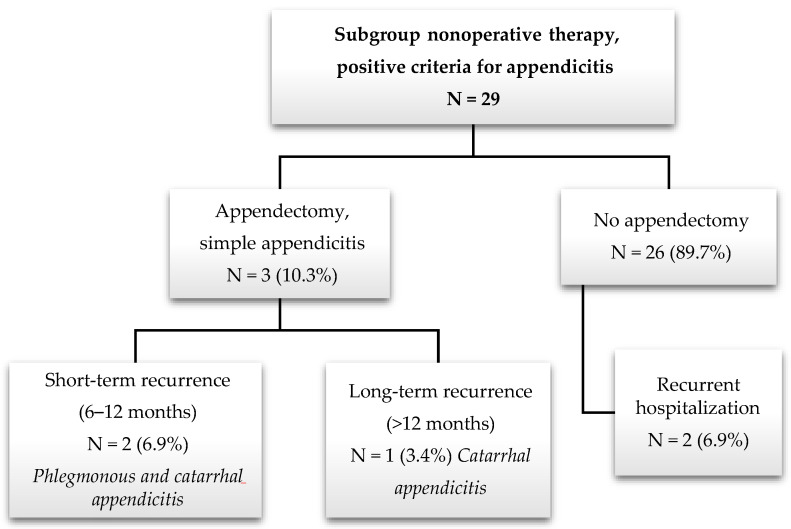
Flowchart of the follow-up of the participants included in the subgroup (n = 29) according to the criteria for acute appendicitis treated nonoperatively. US: ultrasound, AS: Alvarado Score, PAS: Pediatric Appendicitis Score.

The follow-up findings for the subgroup of 29 patients classified as having appendicitis are reported in Figure 4. In summary, 10.3% of these patients underwent secondary appendectomy with histologically confirmed appendicitis recurrence. Two patients presented with short-term recurrence (7.9 and 8.9 months after discharge), and one patient presented with long-term recurrence (23.2 months after discharge). Recurrent hospitalization without appendectomy was reported in two patients (6.9%). Eight patients (2.8%) reported relevant abdominal pain within the first 3 months of conservative therapy.

## 4. Discussion

This descriptive observational study of pediatric patients with suspected simple appendicitis following nonoperative, antibiotic-free management shows that this approach can be feasible and safe in the target population. After a follow-up of at least one year, 6.2% of the patients in the general conservative group underwent appendectomy. In a subgroup of patients classified as having a high probability of having appendicitis, the secondary appendectomy rate was 10.3%. During the follow-up period, no complications were reported.

In our study, patients with suspected uncomplicated appendicitis were admitted and underwent clinical examination, often combined with laboratory and imaging evaluations (especially US), observation and inflammation control. Patients received supportive care, which included reduced enteral intake, intravenous fluids, analgesics, and enemas. A comparison of patient data between the conservative and surgical groups could not support a diagnosis of appendicitis in the nonoperative cohort, as most of the parameters were significantly different. Generally, patients in the operative group more often had clinical appendicitis signs and symptoms, elevated inflammatory marker levels, or sonographic appendicitis signs. It is possible that patients treated nonoperatively may have presented a milder form of appendicitis or even another abdominal condition that also resolved nonoperatively. Based on this fact, the cohort of patients treated nonoperatively might have included many individuals without acute or subacute appendicitis. Nevertheless, the most analyzed parameters were comparable between the group with histologically confirmed appendicitis and the subgroup classified as having a high probability of appendicitis, revealing significant differences only for fever and neutrophilia. In our analysis, negative and simultaneous appendectomies accounted for 5.9% of the procedures, which is comparable with the findings in the literature [32]. We decided to exclude these patients from the analysis because the data from the conservative group should be compared with the data from patients with confirmed appendicitis.

Even though pediatric appendicitis is a common condition, its diagnosis remains challenging, especially retrospectively and following a nonoperative approach. Several scoring systems have been developed to assist physicians in the treatment of suspected appendicitis. In the present study, we chose AS and PAS to label patients in a subgroup at high risk of appendicitis due to their practicability since both scores combine routine clinical and biochemical parameters. Thus, these scores have been the subject of previous validation studies in the pediatric population [28,29,33,34]. If the scores are used alone, they can be helpful for excluding appendicitis in an emergency setting (area under the curve [AUC] of 84% for Alvarado ≤ 3 and PAS ≤ 2) [34]. A previous prospective study reported a sensitivity of 90.4% and a specificity of 91.2% for AS and a sensitivity of 88.1% and a specificity of 98.2% for PAS at a cutoff point of 6 [28]. The combination of intermediate scores (5–8 for AS and 4–7 for PAS) with US suggesting appendicitis increased the sensitivity to 93.3% and 97.2% for AS and PAS, respectively, while the specificity reached 100% for AS and slightly decreased to 97.6% for PAS [28,33]. Another prospective evaluation of the proposed diagnostic tool reported a diagnostic accuracy of 94% (confidence interval [CI] 91–97%) [29]. A cutoff of 6 mm for appendix diameter has also been previously described as indicative of a sonographic appendicitis sign, especially when combined with relevant clinical findings [17,35,36].

Biochemical and immunological inflammatory cascade analyses, as well as observations of the natural history and epidemiology of this disease, indicate that complex and simple appendicitis might present a distinct pathophysiology [19,21,37,38]. Spontaneous healing of mild forms of acute uncomplicated appendicitis has been observed and described, but no antibiotic-free indications or therapeutic regimens have been established [3,19,39]. A relevant number of studies have evaluated conservative therapy for acute uncomplicated appendicitis with antibiotics in children and adults, comparing diagnostic and therapeutic outcomes with operative therapy and confirming the feasibility of a nonoperative approach with antibiotic therapy [4,6,9,12,40]. A recent meta-analysis reported that nonoperative treatment with antibiotics is safe, although recurrence occurred in 32% of the patients after one year [9]. Ohba et al. proposed in a prospective trial combining B-mode and Doppler US findings for treating pediatric appendicitis conservatively without antibiotics if the appendix wall perfusion was normal or increased [17]. The study reported initial spontaneous improvement, with a recurrence rate of up to 27% after discharge. Decreased blood flow with irregularity or loss of the submucosal layer is indicative of necrotic change, and these patients underwent primary appendectomy [17]. In a randomized clinical trial including only adult patients, treatment failure rates after conservative therapy with or without antibiotics (20.7% vs. 23.4%, median follow-up time of 19 months) were not significantly different [18]. Salminen et al. reported similar outcomes after comparing antibiotics vs. placebo in adults [41]. There is still a lack of similar evaluations in children.

Generally, our secondary appendectomy rates (6.2%, 4.4% histologically confirmed, for the general conservative study population vs. 10.3% for the subgroup) during follow-up are low compared to those in the literature. These comparisons must be performed carefully, as different studies have different inclusion criteria for diagnosis and management, and most of them used antibiotics [6,9,17]. Treatment failure was evaluated only after discharge in our population, as patients who underwent initial observation and then appendectomy were considered operative. One patient underwent appendectomy 5 days after initial discharge with pain improvement and subsequent early pain recurrence, presenting histopathological phlegmonous appendicitis. For this patient, early failure of nonsurgical therapy could be confirmed. Nevertheless, the safety of the presented therapy is illustrated by the fact that no patients presented with complications during follow-up. Generally, 93.8% of the complete conservative study population (212 of 226 patients) and 89.7% of the subgroup (26 of 29 patients) did not undergo appendectomy during the follow-up period. Since the diagnosis of this population could not be histologically confirmed and the inclusion criteria did not include CT or detailed US findings, it remains unclear which patients truly presented with appendicitis. The diagnosis might have also been another abdominal condition that healed spontaneously or was resolved by supportive care.

The advantages of a nonoperative therapy consist of preserving the appendix vermiformis and its immune function [42,43], avoiding operation- and anesthesia-related risks and negative appendectomies, saving medical resources and, in the case of the nonuse of antibiotics, preventing the generation of further bacterial resistance. Arguments against conservative approaches include possible pain relapse, rehospitalization, appendicitis recurrence, possible complications due to delayed surgery such as perforation [7,9,20,37,44], and undetected neoplasia (for which the incidence of carcinoid tumors in the appendix is approximately 0.3–0.49%) [45,46,47].

Based on our results, especially regarding the absence of complicated cases during follow-up and the low recurrence rate, supported by the current literature and further clinical observations, we developed an internal clinical pathway for the management of upcoming pediatric patients with suspected appendicitis presenting to the emergency department (Appendix A). The proposed algorithm was used as a basis for patient management and prospective data collection for further research with a larger dataset of patients and variables, including the use of machine learning for predicting the diagnosis, management, and severity of pediatric appendicitis [25,48]. The most important predictors for all target outcomes were abdominal guarding, appendix diameter, white blood cell count, neutrophil percentage, and CRP at entry [25].

Our study is limited primarily by its retrospective design, small sample size, retrospective diagnoses, and criteria for allocating patients to the general conservative group. Further limitations included incomplete documentation of patient charts and missing variables, which also limited the possibility of defining predictors. Poorly documented variables, such as abdominal guarding, fecalith, and appendix wall perfusion on US, did not allow for the analysis of those parameters in the current study. In addition, due to missing data, proper assessment of the appendicitis scores of the surgical group was also not feasible. Selection bias between the two groups was also observed, as patients who presented with a more severe disease course were more frequently selected for a primary operation. Thus, the lack of histological examination of conservative patients precludes the confirmation of an appendicitis diagnosis, unlike for operated patients. The probable inclusion of patients without appendicitis in the conservative group limited the validity of the results. However, the combination of available scores and US findings seems to be a valid option for diagnostic assessment. Thus, only patients with suspected appendicitis admitted to the pediatric surgery unit were included, not those that had been discharged after presenting to the emergency department. Moreover, the strength of the present study was the low loss to follow-up rate, which allows for a wide estimation of rehospitalization and the recurrence of (suspected) uncomplicated appendicitis. In addition, few publications on this topic exist, especially in the pediatric population.

Further research should focus on identifying, during initial assessment, which children and adolescents might adequately respond to a nonoperative approach—with or without antibiotics—and which patients should be referred for surgery. Based on our findings, conservative, antibiotic-free management is feasible in selected patients. Identifying these patients at admission or during the initial observation period can reduce surgical and anesthesiologic risks, postoperative complications, and negative appendectomy rates. Thus, the goal of further research should be the early differentiation of different forms of appendicitis (simple vs. complicated) and their course (self-limiting vs. progressive disease) to support the basis of an individual therapeutic approach.

## 5. Conclusions

In conclusion, a nonoperative, antibiotic-free approach for children and adolescents presenting with mild signs and symptoms and laboratory and US findings suggesting uncomplicated appendicitis may be safely applied in a hospital setting, including active observation. However, further prospective and randomized trials are needed to establish a consistent recommendation regarding diagnosis and management and to differentiate patients who can be conservatively treated with or without antibiotics from those who should be referred for surgery.

## Figures and Tables

**Table 1:** Appendicitis Scores.

Alvarado Score (AS)		Pediatric Appendicitis Score (PAS)	
Diagnostic Criteria	Value	Diagnostic Criteria	Value
Migration of pain	1	Migration of pain	1
Anorexia/Ketones in urine	1	Anorexia	1
Nausea/Vomiting	1	Nausea/Emesis	1
Tenderness in the right lower quadrant	2	Tenderness in the right lower quadrant	2
Rebound pain	1	Cough/Percussion/Hopping tenderness	2
Elevation of temperature (≥37.3 °C)	1	Fever (≥38 °C)	1
Leukocytosis (≥10.000/µL)	2	Leukocytosis (≥10.000/µL)	1
Neutrophilia (≥75%)	1	Neutrophilia (≥75%)	1

AS, points: 1–4, low appendicitis probability; 5–8, intermediate risk (5–6, possible appendicitis; 7–8, probable appendicitis); 9–10, high risk [26,28]. PAS, points: 1–3, low risk; 4–7, intermediate risk; 8–10, high risk [27,28,29].

**Table 2:** Inclusion and exclusion criteria.

Included	Excluded
Hospital admission to the pediatric surgery unit due to abdominal pain and suspected appendicitis.	Outpatients.
Age < 18 years.	Patients who have undergone appendectomy before.
Surgical group: histological uncomplicated appendicitis (catarrhal, phlegmonous, or chronic).	Surgical group: negative and incidental appendectomy, complicated appendicitis (gangrenous, abscess, or perforated).
	Another relevant diagnosis as the cause of abdominal pain (intussusception, pneumonia, or ovarian torsion) or relevant comorbidities (neoplasia or chronic intestinal disease, such as Crohn’s disease and ulcerative colitis).
	Conservative group: antibiotics during hospitalization; loss to follow-up.

**Table 3:** Patient group characteristics of the study population.

Parameters *	Appendectomy N = 139	Nonoperative Treatment N = 226	*p*-Value ^§^	Subgroup Nonoperative Treatment N = 29	*p*-Value ^§§^
Demographics					
Sex			0.161		0.686
Male	75 (54%)	104 (46%)		17 (59%)	
Female	64 (46%)	122 (54%)		12 (41%)	
Age in years (mean)	10.8 (SD 3.4)	10.6 (SD 3.3)	0.501	10 (SD 2.3)	0.249
Inflammatory parameters					
Leukocytosis (≥10/nL)	104 ^b^ (75%)	108 ^a^ (48%)	**0.000**	23 (79%)	0.812
CRP elevated (>5 mg/L)	92 ^c^ (68%)	73 (32%)	**0.000**	14 (48%)	0.057
Neutrophilia (≥75%)	97 ^e^ (73%)	66 ^d^ (30%)	**0.000**	15 (52%)	**0.044**
Clinical parameters					
Nausea or vomiting (yes)	69 ^f^ (57%)	94 ^a^ (42%)	**0.010**	17 (59%)	1.000
RLQ tenderness (yes)	138 (99%)	197 ^a^ (88%)	**0.000**	29 (100%)	1.000
Rebound pain (yes)	56 ^h^ (50%)	42 ^g^ (20%)	**0.000**	9 ^o^ (32%)	0.137
Fever (temp. ≥ 38 °C)	64 (46%)	40 (18%)	**0.000**	7 (24%)	**0.038**
Ultrasound records					
Free intraabdominal fluid ^+^	47 ^j^ (37%)	46 ^i^ (21%)	**0.002**	6 ^o^ (21%)	0.129
Visible appendix ^+^	69 ^l^ (54%)	61 ^k^ (28%)	**0.000**	29 (100%)	**0.000**
Appendix diameter in mm (mean)	7.6 ^n^ (SD 1.6)	6.6 ^m^ (SD 2.4)	**0.009**	7.5 (SD 2.2)	0.835
Length of hospital stay					
Days (mean)	4.5 (SD 2.7)	1.9 (SD 0.8)	**0.000**	2.1 (SD 0.9)	**0.000**

* Data are displayed as the number of patients and percentages unless stated otherwise. ^§^ Appendectomy vs. nonoperative treatment. ^§§^ Appendectomy vs. subgroup nonoperative treatment. ^+^ Presence of. RLQ: Right lower quadrant. SD: Standard deviation. Documented cases: ^a^ N = 224; ^b^ N = 138; ^c^ N = 136; ^d^ N = 221; ^e^ N = 133; ^f^ N = 122; ^g^ N = 215; ^h^ N = 112; ^i^ N = 218; ^j^ N = 127; ^k^ N = 219; ^l^ N = 128; ^m^ N = 52; ^n^ N = 55; ^o^ N = 28. Significant *p*-values are marked in bold.

**Table 4:** Primary appendectomies and histopathology.

Primary Appendectomy Group	N = 139
Phlegmonous appendicitis	106 (76.3%)
Catarrhal appendicitis	20 (14.4%)
Chronic appendicitis	13 (9.3%)

Phlegmonous: Mucosal ulceration; neutrophilic infiltration of the submucosa, muscularis propria, and serosa layers; possibly microabscesses of the wall; and thrombosis/hemorrhage. Catarrhal: Focal mucosal erosions, inflammatory infiltrate in the submucosal layer, and edema. Chronic: infiltration of the lamina propria by lymphocytes, histiocytes, and plasma cells; lymphoid tissue hyperplasia; and fibrosis (local pathology reports) [16,30,31].

**Table 5:** Follow-up: Secondary appendectomies and histopathology.

Secondary Appendectomy Group	N = 14
Phlegmonous appendicitis	4 (28.6%)
Catarrhal appendicitis	3 (21.4%)
Chronic appendicitis	3 (21.4%)
Normal appendix	2 (14.3%)
No histology available *	2 (14.3%)

* According to patient information, uncomplicated appendicitis.

**Figure A1:**
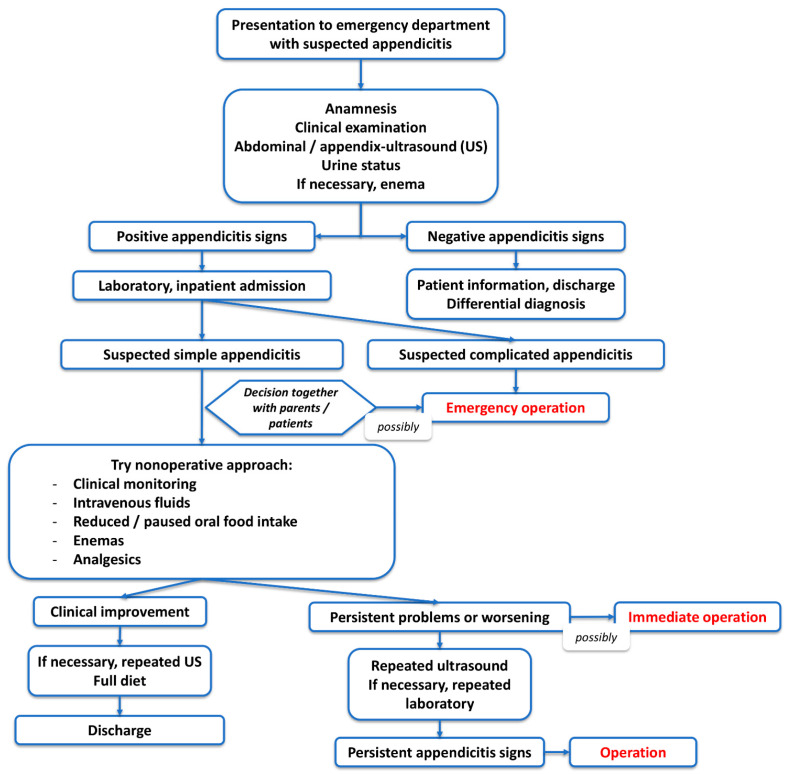
Flowchart of the management of pediatric patients with suspected appendicitis. Department of Pediatric Surgery and Pediatric Orthopedics, Hospital St. Hedwig of the Order of St. John of God, University Children’s Hospital Regensburg (KUNO), Regensburg, Germany [25,48].

## Data Availability

The data presented in this study are available from the corresponding author upon request. The data are not publicly available due to ethical and privacy reasons.
